# A Novel Intraluminal Microtube Anastomosis System (IMAS) for Rapid Rat Microvascular Reconstruction: Development and Experimental Evaluation

**DOI:** 10.3390/jcm15218277

**Published:** 2026-10-24

**Authors:** Sam-Youl Yoon

**Affiliations:** Department of Surgery, Sanggye Paik Hospital, Inje University College of Medicine, Seoul 01757, Republic of Korea; cdgx287@gmail.com; Tel.: +82-2-950-1114; Fax: +82-50-7077-4445

**Keywords:** microvascular anastomosis, intraluminal stent, intimal anchoring, rat abdominal aorta, microsurgery, 3D printing

## Abstract

**Background/Objectives**: Conventional interrupted microvascular anastomosis is technically demanding and time-consuming. We developed an intraluminal microtube anastomosis system (IMAS) in which circumferential anchoring ridges on the outer surface mechanically retain each arterial end while two external circumferential ligatures secure the reconstruction. This study compared IMAS with conventional interrupted microsurgical anastomosis in a rat infrarenal abdominal aortic model. **Methods**: Forty male Sprague Dawley rats (250–300 g) were allocated to IMAS (*n* = 20) or conventional interrupted microsurgical anastomosis (*n* = 20). Random-sequence generation and allocation concealment were handled by an animal-experiment assistant. All formal procedures were performed by the same surgeon, who was proficient in conventional microsuturing and had completed 10 pilot IMAS procedures before the study. Anastomosis time was measured from complete aortic transection to restoration of flow after clamp removal. Patency was assessed at 1 week and 1 month by color Doppler ultrasonography, with terminal gross examination at 1 month. The study was exploratory and was not powered as a patency non-inferiority or equivalence study. **Results**: Mean anastomosis time was 5 min 30 s ± 60 s with IMAS and 20 min 32 s ± 75 s with conventional suturing (*p* < 0.001). The between-group mean difference (conventional minus IMAS) was 902 s (95% CI, 859–945 s), corresponding to an approximately 73% reduction. Patency was 19/20 (95%) in both groups at 1 week (*p* = 1.000) and 18/20 (90%) in both groups at 1 month (*p* = 1.000). No gross device migration, aneurysmal dilatation, or anastomotic leakage was observed in patent IMAS-treated vessels at necropsy. **Conclusions**: In this exploratory rat model, IMAS substantially shortened reconstruction time. The observed short-term patency proportions were the same in the two groups, but the study was not sufficiently powered to establish equivalence or non-inferiority in patency. Histological response, lumen stenosis, quantitative flow, mechanical strength, material biocompatibility, and longer-term outcomes require further evaluation.

## 1. Introduction

Microvascular anastomosis is a fundamental technique in reconstructive surgery, transplantation, vascular surgery, and experimental vascular research. Restoration of blood flow through a small-caliber vessel requires accurate approximation of the transected vessel ends while preserving luminal continuity and minimizing leakage, thrombosis, stenosis, and mechanical failure. Conventional interrupted hand-sewn anastomosis remains one of the most widely used techniques because individual sutures allow precise adjustment of vessel-wall approximation and can provide reliable long-term patency when performed appropriately. However, successful microsurgical anastomosis requires substantial technical skill, particularly when the vessel diameter is small, and the operative field must be manipulated under high magnification [1,2].

The conventional interrupted technique involves repeated passage of a fine microsurgical needle through the vessel wall, followed by placement and tying of multiple sutures. The operator must maintain appropriate spacing between sutures, avoid inadvertent incorporation of the opposite vessel wall, prevent excessive tension, and preserve the luminal geometry of the reconstructed vessel. These requirements make the procedure technically demanding and dependent on operator experience. Furthermore, repeated needle manipulation and knot tying contribute to the overall duration of vascular clamping and reconstruction.

Anastomosis time is relevant not only to surgical efficiency but also to experimental reproducibility. In animal models involving vascular reconstruction or transplantation, variation in operative technique and duration can introduce additional variability into the experimental procedure. A reconstruction technique that requires fewer technically demanding steps may therefore be valuable as an experimental platform by simplifying the procedure and potentially reducing operator-dependent variation.

Various approaches have been proposed to simplify small-vessel reconstruction. Continuous suturing can reduce the number of knots required and shorten anastomosis time compared with conventional interrupted suturing in some settings. Nevertheless, continuous anastomosis remains dependent on precise microsurgical needle placement and appropriate control of suture tension [2,3,4]. Errors in tension or vessel-wall approximation may influence the geometry of the reconstructed segment.

Cuff techniques provide another method for reducing the number of microsutures required. These methods have been used particularly in experimental graft and transplantation models, where a vessel can be placed over a cuff and secured externally. Cuff-based reconstruction can shorten vascular clamping and reconstruction times; however, the procedure generally requires manipulation or eversion of the vessel wall and may not be readily applicable to every form of end-to-end arterial reconstruction [5,6].

Several intraluminal or sutureless vascular reconstruction strategies have been described. Published approaches include hollow intraluminal stents used primarily to maintain luminal geometry during photochemical tissue bonding, expandable shape-memory alloy stents combined with adhesive fixation, modified micro-stents, and temporary intravascular stents used to prevent luminal collapse during cyanoacrylate bonding [7,8,9,10]. These systems demonstrate the feasibility of reducing dependence on multiple interrupted sutures, but their principal fixation mechanisms rely on luminal support, radial expansion, adhesive bonding, or combinations of these strategies.

IMAS was developed as a different mechanical concept: a permanent cylindrical microtube with circumferential anchoring ridges on its outer surface. Each transected arterial end is advanced over one side of the microtube until the intimal surface engages the ridge; a circumferential ligature is then tied externally over the vessel. The ridge is intended to resist longitudinal withdrawal of the vessel end, while the external ligature compresses the vessel onto the device. Thus, the ridge functions as a retention element rather than as a depth marker or simple luminal spacer.

In the prior systems cited above, we did not identify an in vivo evaluation of the same bilateral ridge-and-ligature retention principle. IMAS also differs from cuff techniques because vessel eversion over an external cuff is not required. These distinctions are procedural as well as mechanical: end-to-end reconstruction is completed with two circumferential ligatures and without multiple transmural interrupted sutures. The expected advantage is therefore a reduction in repetitive needle passage and knot tying while maintaining mechanical resistance to vessel-end withdrawal.

The rat infrarenal abdominal aorta provides a small-caliber arterial model in which complete transection and subsequent end-to-end reconstruction can be directly evaluated. In the present controlled animal study, we compared IMAS-assisted reconstruction with conventional interrupted microsurgical anastomosis in this model. The primary outcome was anastomosis time, and the secondary outcome was postoperative vascular patency.

The objective of this study was to determine whether the IMAS fixation principle could substantially shorten the time required for arterial reconstruction while maintaining short-term vascular integrity and patency. We hypothesized that mechanical retention of both vessel ends using circumferential anchoring ridges and two external ligatures would reduce the technical steps required for reconstruction and thereby shorten anastomosis time compared with conventional interrupted microsurgical suturing.

## 2. Materials and Methods

### 2.1. Study Design and Reporting

This study was a controlled animal experiment comparing two methods of end-to-end reconstruction of the infrarenal abdominal aorta. Forty rats underwent complete aortic transection followed by reconstruction with either IMAS or conventional interrupted microsurgical suturing. The same vessel model, operative exposure, anesthesia, heparinized irrigation, vascular clamps, microscope, and peri-anastomotic workflow were used for both groups.

Animals were allocated in a 1:1 ratio to the IMAS group (*n* = 20) or the conventional group (*n* = 20) using permuted block randomization with a fixed block size of two. For each two-animal block, an animal-experiment assistant used a random-number table to determine which treatment was performed first, and the second animal in the block was assigned to the alternative treatment. This approach maintained balanced allocation between the two groups throughout the experiment. The allocation sequence was prepared and managed by the animal-experiment assistant and was disclosed to the operating surgeon immediately before each procedure.

The primary outcome was anastomosis time. The secondary outcome was postoperative vascular patency at 1 week and 1 month. Immediate restoration of arterial flow after clamp removal was assessed as a procedural confirmation of successful reconstruction rather than as a separate secondary outcome. All operations in both groups were performed by the same surgeon, who was already proficient in conventional microvascular suturing before the study. Before initiation of the formal experiment, the surgeon completed 10 pilot IMAS procedures for device handling and procedural familiarization. These pilot procedures were not included in the 40-animal study cohort or in the formal analyses. The same start and end points were used for procedural timing in both groups.

No prospective effect-size-based sample-size calculation was performed. The cohort of 20 animals per group constituted an exploratory feasibility sample intended to characterize procedural feasibility, estimate the magnitude and variability of the anastomosis-time difference, and provide preliminary short-term patency observations. The sample size was therefore not selected to test equivalence or non-inferiority in patency or to estimate uncommon adverse events with precision.

### 2.2. Device Design and Fabrication

The IMAS was designed as a cylindrical microtube intended for permanent placement within the lumen of the reconstructed artery.

Three-dimensional models were digitally designed using Fusion 360 (version 2.0.3800, 2018; Autodesk Inc., San Rafael, CA, USA) and fabricated using a Rubicon MAS 300 stereolithography printer (Rubicon Co., Ltd., Seongnam, Gyeonggi-do, Republic of Korea) with Anycubic Tough Ultra Resin (Anycubic, Shenzhen, Guangdong, China), a photopolymer resin formulated for 365–405 nm light curing. According to the manufacturer-provided technical specification, the resin has a density of 1.05–1.25 g/cm^3^, a Shore hardness of 71–75 D, a tensile strength of 20–28 MPa, an elongation at break of 70–80%, a flexural strength of 15–25 MPa, a flexural modulus of 350–800 MPa, a linear shrinkage of 5.1–6.2%, and a heat-deflection temperature of 50–55 °C. The product information specifies alcohol as the resin-cleaning solvent. However, the retained experimental record does not document the resin lot number, detailed chemical composition, formal medical-device biocompatibility classification, study-specific post-curing parameters, sterilization method, surface-finishing procedure, or manufacturing tolerances. Dedicated hemocompatibility testing of the implanted resin was not performed; therefore, the present experiment should not be interpreted as establishing material hemocompatibility or long-term blood-contacting safety.

The cylindrical microtube had an inner diameter of 1.6 mm, an outer diameter of 1.8 mm, and an overall length of 8.5 mm. The 8.5 mm length is the verified device dimension used in this study. The central lumen provided a continuous intraluminal flow passage after reconstruction.

Circumferential anchoring ridges were incorporated into the external surface of the device on both sides of the central segment. After a transected arterial end was advanced over the microtube, the ridge contacted the intimal surface. A 10-0 nylon circumferential ligature was then placed externally over the vessel and device. The ligature compressed the vessel wall around the microtube, whereas the ridge provided resistance to longitudinal withdrawal of the vessel end.

The same fixation mechanism was applied independently to the proximal and distal arterial ends. Thus, the completed anastomosis depended on the combined mechanical action of bilateral intraluminal anchoring ridges and two external circumferential ligatures, rather than on multiple transmural sutures, radial stent expansion, or adhesive bonding.

**Figure 1:**
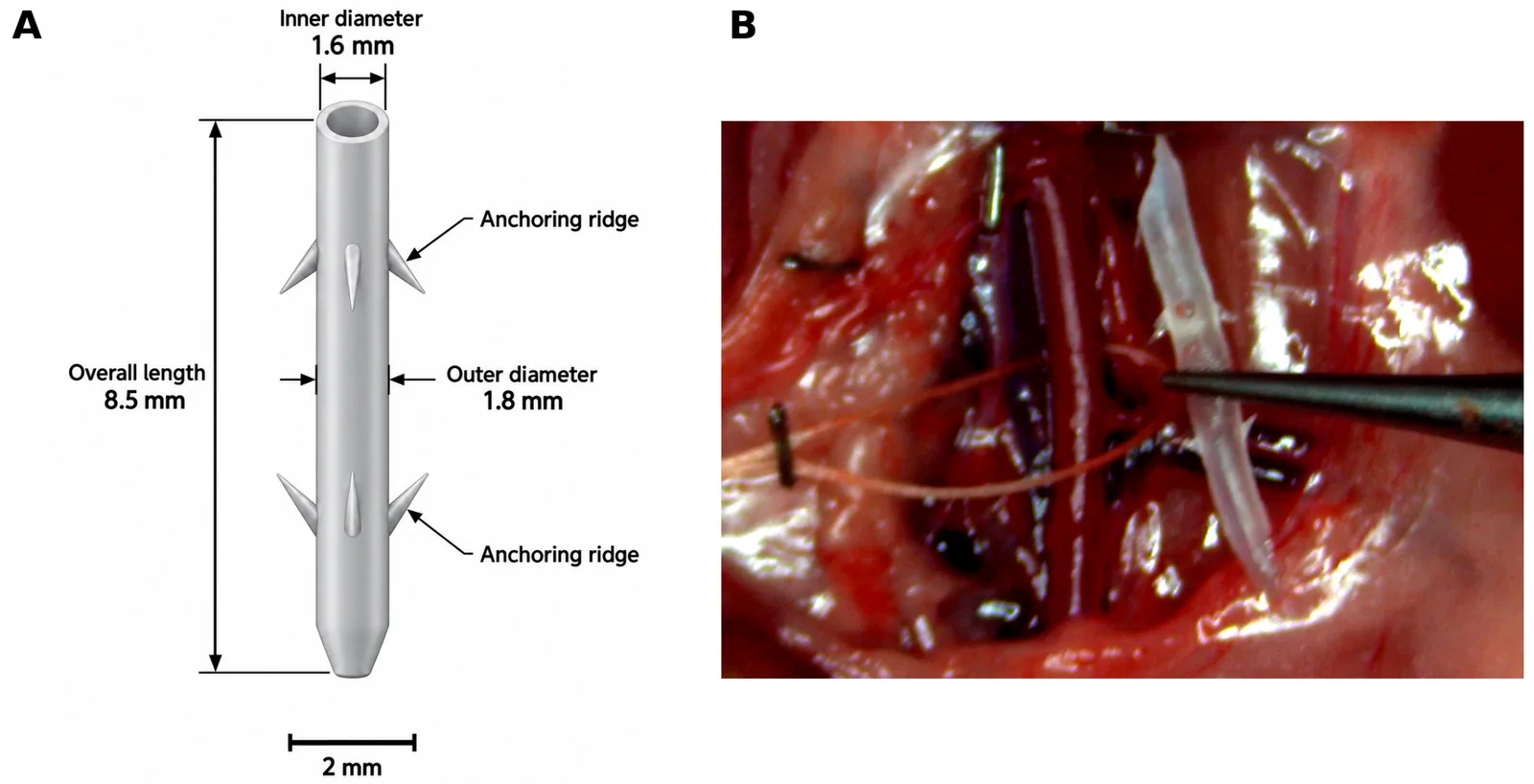
IMAS design, dimensions, anchoring ridges, and intraoperative placement: (**A**) Labeled CAD rendering of the intraluminal microtube anastomosis system showing the central lumen (inner diameter, 1.6 mm), external cylinder (outer diameter, 1.8 mm), overall length (8.5 mm), and bilateral anchoring ridges; scale bar, 2 mm. (**B**) Representative intraoperative view of IMAS-assisted reconstruction of the rat infrarenal abdominal aorta. The microtube is positioned intraluminally within the transected artery and secured by external circumferential ligation. After implantation, the anchoring ridges are covered by the vessel wall and are therefore not directly visible in the operative photograph.

The microtube was not removed and remained within the reconstructed arterial segment throughout the 1-month follow-up period. Before the formal study, preoperative ultrasonographic screening of the abdominal aorta was performed in male Sprague Dawley rats weighing 250–300 g to estimate vessel caliber and guide device sizing. Based on this screening, an IMAS with an inner diameter of 1.6 mm and an outer diameter of 1.8 mm was considered likely to provide an appropriate fit. During the 10 pilot IMAS procedures, adjacent device sizes were also tested, with both the inner and outer diameters reduced or increased by 0.2 mm (1.4/1.6 mm and 1.8/2.0 mm, inner/outer diameter, respectively). Among these sizes, the 1.6/1.8 mm device provided the most suitable practical fit during the pilot procedures and was therefore selected for the formal 40-animal experiment. The ultrasonographic screening was used for device sizing rather than as a prespecified quantitative outcome; therefore, group-level vessel-diameter data were not included in the formal analysis. A labeled CAD rendering showing the device dimensions and anchoring ridges is provided in Figure 1A, and representative intraoperative placement is shown in Figure 1B.

### 2.3. Animals, Housing, and Ethics

Forty male Sprague Dawley rats weighing 250–300 g were included; 20 underwent IMAS reconstruction, and 20 underwent conventional interrupted microsurgical anastomosis. Rats were obtained at 6 weeks of age from Daehan Bio Link Co., Ltd., Laboratory Animal Division (Eumseong-gun, Republic of Korea). The animals had specific pathogen-free (SPF) microbiological status and were housed in a barrier facility. After an acclimatization period of 1–2 weeks, the experiments were performed at 7–8 weeks of age. All 40 allocated animals were included in the reported outcome analyses.

The experimental protocol was approved by the Institutional Animal Care and Use Committee (IACUC No. HMC-2019-1-0612-17) and was conducted in accordance with applicable institutional guidelines for animal experimentation.

Postoperative analgesia was administered in accordance with the IACUC-approved protocol. To reduce postoperative pain and inflammation, all animals received carprofen (5 mg/kg per dose) by injection twice daily after surgery. No antibiotics or systemic anticoagulants were administered; intraoperative local heparinized saline was used as described below. After surgery, an animal-experiment assistant monitored each animal twice daily for general condition and activity, behavior, food intake, and signs of surgical-site infection. At the end of the study, animals were euthanized by carbon dioxide inhalation. Male rats were selected to reduce experimental complexity and anatomical variability and to facilitate standardized microsurgical exposure in this feasibility model. No animals were excluded from the reported analyses after allocation. A detailed prespecified definition of humane endpoints and a separate list of prespecified exclusion criteria were not documented in the retained protocol.

### 2.4. Operative Preparation

All procedures in both groups were performed by the same surgeon under an operating microscope (Olympus Corporation, Tokyo, Japan). The surgeon was already proficient in conventional microvascular suturing and had completed 10 pilot IMAS procedures before the formal experiment. No separate surgeons were assigned to the two techniques. The operating surgeon could not be blinded to technique because the reconstruction methods were visibly different.

General anesthesia was induced and maintained with inhaled 2% isoflurane using the SomnoSuite^®^ Small Animal Anesthesia System (Kent Scientific Corp., Torrington, CT, USA).

A midline laparotomy was performed. The infrarenal abdominal aorta was exposed and isolated, and sufficient vessel length was mobilized to permit clamping, complete transection, and end-to-end reconstruction. These preparatory steps were performed in the same manner for both groups.

The vessel was locally irrigated with heparinized saline (100 IU/mL), and proximal and distal vascular control was obtained using microvascular clamps (S&T AG, Neuhausen, Switzerland). The aorta was then completely transected, producing two free arterial ends.

Procedural timing began only after complete transection. Therefore, anesthesia induction, laparotomy, aortic exposure, heparinized irrigation, and clamp placement occurred before the timed interval and did not contribute to the reported anastomosis time. Timing ended when the clamps had been removed, and arterial flow was restored; wound closure and postoperative care occurred after the timed interval.

### 2.5. IMAS Anastomosis

The IMAS procedure followed a fixed sequence. First, one end of the cylindrical microtube was introduced into the proximal arterial lumen. Second, the proximal arterial end was advanced over the outer surface of the microtube until its intimal surface engaged the circumferential anchoring ridge.

Third, the distal arterial end was advanced over the opposite end of the microtube until it engaged the contralateral anchoring ridge. The arterial ends were not everted during placement, distinguishing the maneuver from a conventional cuff technique.

Fourth, a 10-0 nylon circumferential ligature (Ethilon; Ethicon Inc., Somerville, NJ, USA) was placed around the proximal arterial segment overlying the device. Fifth, a second 10-0 nylon circumferential ligature was placed around the distal arterial segment. No additional interrupted sutures were placed through the opposing vessel walls.

The two ligatures compressed the arterial wall onto the microtube, while the underlying ridges resisted longitudinal withdrawal. After both vessel ends had been secured, the microvascular clamps were removed.

**Figure 2:**
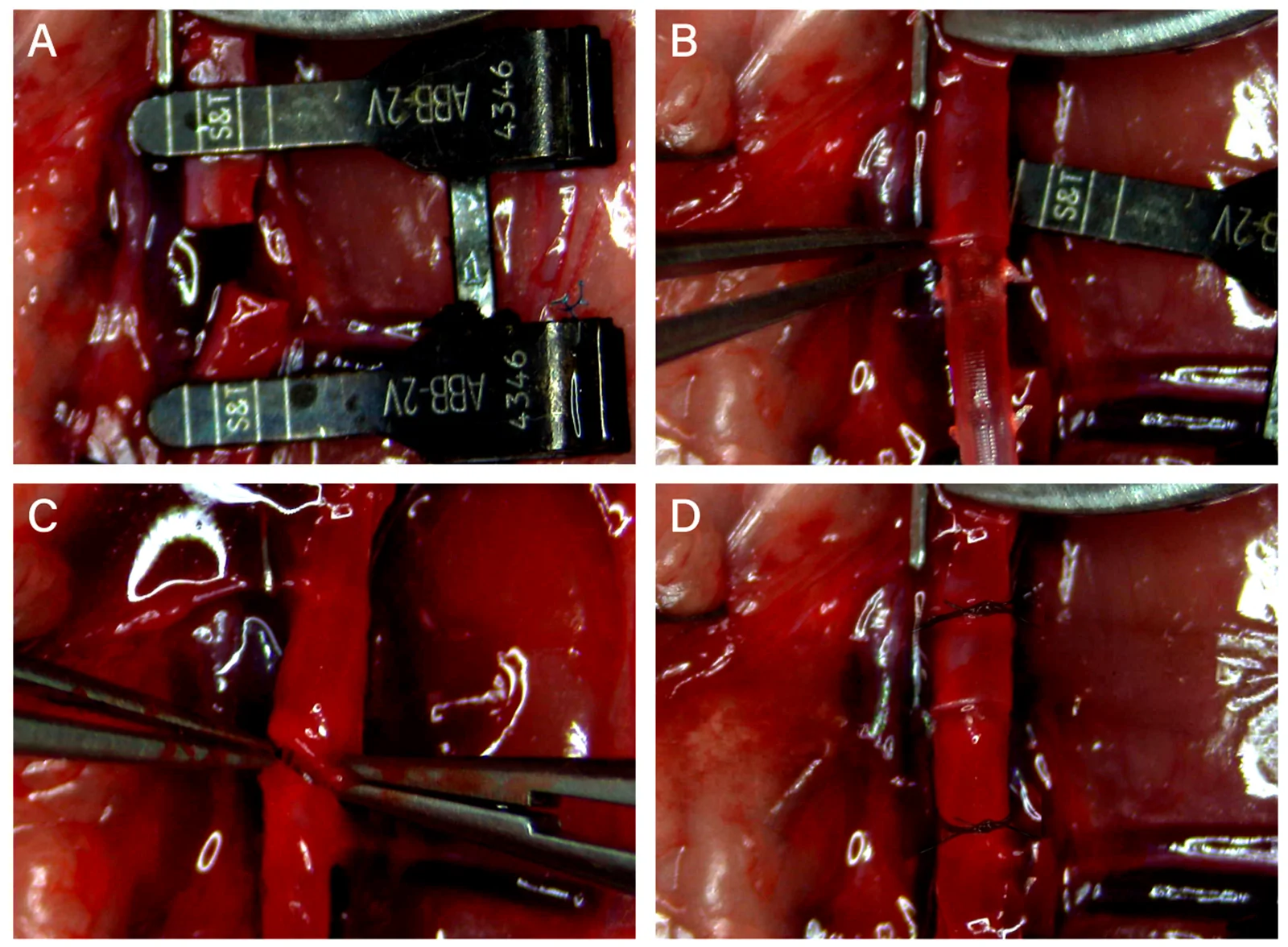
Stepwise IMAS-assisted end-to-end reconstruction of the rat infrarenal abdominal aorta: (**A**) After common operative preparation, proximal and distal microvascular clamps are in place, and the aorta has been completely transected; this event marks the start of procedural timing. (**B**) The proximal arterial end is advanced over one side of the microtube until the vessel engages the anchoring ridge. (**C**) The distal arterial end is advanced over the opposite side of the microtube while the two vessel ends are aligned. (**D**) Final reconstruction after separate proximal and distal 10-0 nylon circumferential ligatures have secured the vessel to the microtube; the clamps are then removed, and restored arterial flow marks the end of timing.

Restoration of arterial flow and obvious anastomotic leakage were assessed visually immediately after clamp removal. The timed interval ended when flow was restored (Figure 2).

### 2.6. Conventional Interrupted Microsurgical Anastomosis

The conventional group underwent the same anesthesia, exposure, heparinized irrigation, clamping, and complete aortic transection described above. After transection, the proximal and distal arterial ends were aligned under microscopic visualization.

Individual 10-0 nylon sutures (Ethilon; Ethicon Inc.) were then passed through the opposing vessel walls and tied sequentially around the circumference until the arterial ends were completely approximated and the anastomotic line was sealed. Unlike IMAS, this technique required repeated transmural needle passage, suture placement, and knot tying.

After completion of the interrupted anastomosis, the microvascular clamps were removed, and restoration of arterial flow and obvious leakage were assessed visually. The same timing endpoint used in the IMAS group was applied.

Thus, the two groups differed principally in the reconstruction steps performed between complete transection and reperfusion: IMAS used intraluminal ridge-based retention plus two circumferential ligatures, whereas the control technique used repeated interrupted transmural sutures.

### 2.7. Outcomes and Follow-Up

Anastomosis time was defined identically in both groups as the interval from complete transection of the abdominal aorta to restoration of arterial flow following removal of the microvascular clamps. This interval intentionally excluded the common pre-transection preparation and post-reperfusion closure steps.

A single assistant measured all procedure times with a handheld stopwatch. Because the reconstruction technique was directly visible, the timing observer was not blinded to group assignment. Video-based independent timing was not performed.

The use of identical start and end events reduced ambiguity in the timing definition, but the non-blinded handheld measurement and the absence of video adjudication are potential sources of measurement bias.

Immediate restoration of arterial flow was assessed visually after clamp removal as confirmation of successful completion of the reconstruction. Postoperative vascular patency, the predefined secondary outcome, was assessed at 1 week and 1 month using color Doppler ultrasonography (SONON 300L; HILSERION Co., Ltd., Seoul, Republic of Korea). The ultrasound examiner was blinded to group assignment during outcome assessment and was not informed whether an animal had undergone IMAS or conventional interrupted suturing. Because both groups had the same abdominal incision, group allocation was not apparent from external inspection. During ultrasonography, the examiner assessed the presence or absence of color Doppler flow across the reconstructed arterial segment and compared the luminal caliber of the proximal and distal abdominal aorta. Patency was defined as detectable color Doppler flow traversing the reconstructed segment. Blood-flow velocity was not measured, and no quantitative percentage stenosis was calculated from ultrasonography.

At 1 month, Doppler ultrasonography was followed immediately by necropsy in all animals. Gross examination included a two-forceps compression maneuver to assess collapse of the distal aorta beyond the reconstructed segment. The abdominal aorta was then opened longitudinally to inspect the luminal surface and assess gross intraluminal narrowing or stenosis. In the IMAS group, device position, visible displacement, aneurysmal dilatation, and anastomotic leakage were also evaluated. Thus, the 1-month Doppler findings were cross-checked against immediate gross examination. The necropsy assessment of luminal narrowing was qualitative rather than a calibrated percentage-stenosis measurement.

Serial Doppler assessment and terminal gross examination therefore provided binary patency information together with qualitative assessment of proximal-distal luminal caliber and gross luminal narrowing. However, blood flow velocity, flow volume, and quantitative percentage stenosis were not measured, and microscopic biological responses of the vessel wall were not assessed.

**Figure 3:**
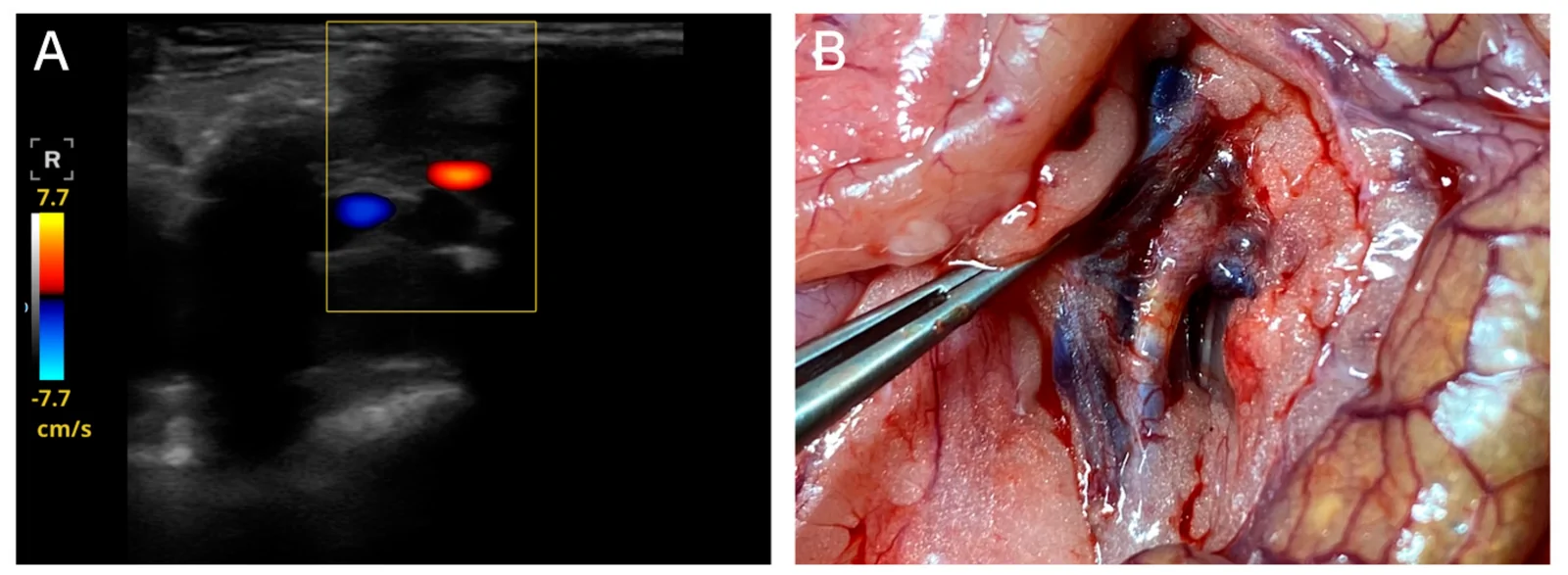
Patency assessment at follow-up: (**A**) Representative color Doppler ultrasonography at 1 month after reconstruction. The red color signal represents flow in the abdominal aorta across the reconstructed segment, whereas the adjacent blue signal corresponds to venous flow in the inferior vena cava. Scheduled Doppler examinations were performed at both 1 week and 1 month; only the representative 1–month image is shown here. The ultrasound examiner was blinded to group assignment and assessed the presence of flow and the relative luminal caliber of the proximal and distal aorta; flow velocity was not measured. (**B**) Gross necropsy view at 1 month showing the reconstructed abdominal aortic segment and retained IMAS in situ. Ultrasonography was followed immediately by necropsy; gross examination included a two-forceps compression maneuver to assess distal aortic collapse, and the aorta was opened longitudinally to inspect the luminal surface for gross narrowing or stenosis.

Histological evaluation was not performed; therefore, endothelial injury, inflammation, thrombus organization, fibrosis, and neointimal hyperplasia were not quantified. Mechanical endpoints such as burst pressure or tensile pullout strength were also not measured (Figure 3).

### 2.8. Statistical Analysis

Statistical analyses were performed using IBM SPSS Statistics for Windows, version 25.0 (IBM Corp., Armonk, NY, USA).

Continuous data are presented as mean ± standard deviation. Anastomosis times were compared using an independent-samples *t*-test. In addition to the *p* value, the between-group mean difference is reported with a 95% confidence interval.

Categorical patency outcomes were compared using a two-sided Fisher’s exact test. This exact test was selected because patency failures were rare (one or two non-patent vessels per group at the scheduled follow-up points), producing sparse 2 × 2 contingency tables. Fisher’s exact test provides an exact conditional test and does not rely on the large-sample chi-square approximation used by Pearson’s chi-square test. A two-sided *p* value < 0.05 was considered statistically significant. These comparisons were exploratory; no statistical test of equivalence or non-inferiority was performed.

## 3. Results

### 3.1. Procedural Completion and Anastomosis Time

All 40 animals underwent the assigned abdominal aortic reconstruction, completed the scheduled follow-up assessments, and underwent 1-month necropsy. No intraoperative deaths were recorded.

Mean anastomosis time was 5 min 30 s ± 60 s in the IMAS group and 20 min 32 s ± 75 s in the conventional group (*p* < 0.001). The mean difference, expressed as conventional minus IMAS time, was 902 s (15 min 02 s; 95% CI, 859–945 s [14 min 19 s–15 min 45 s]).

Using the group means, IMAS reduced anastomosis time by approximately 73% relative to conventional interrupted microsurgical reconstruction.

Because the timed interval began after all common preparatory steps and ended at reperfusion in both groups, the measured difference reflects the reconstruction-specific steps performed between transection and restoration of flow under this single-surgeon experimental setting.

### 3.2. Vascular Patency and Gross Findings

At 1 week, vascular patency was observed in 19 of 20 animals (95%) in the IMAS group and 19 of 20 animals (95%) in the conventional group (two-sided Fisher’s exact *p* = 1.000). At 1 month, patency was observed in 18 of 20 animals (90%) in each group (*p* = 1.000). All 20 animals per group were evaluated at both scheduled follow-up points (Table 1).

Thus, one non-patent vessel per group was identified at 1 week, and two non-patent vessels per group were present by 1 month. None of the four animals with aortic occlusion at 1 month exhibited gross hindlimb paresis or paralysis during twice-daily postoperative observations. Although hindlimb motor impairment was considered a potential clinical consequence of aortic occlusion, no clinically apparent motor deficit was observed. A formal neurological scoring system was not used.

At the 1-month terminal necropsy, all 40 animals underwent gross examination of the reconstructed abdominal aorta. All 20 implanted devices in the IMAS group were examined. Among the 18 patent IMAS-treated vessels, no gross device migration, aneurysmal dilatation, or anastomotic leakage was observed. In the two occluded IMAS-treated vessels, gross examination demonstrated thrombotic occlusion of the reconstructed arterial segment.

Gross luminal narrowing was assessed during necropsy, but percentage stenosis was not quantitatively measured. Histological analysis was not performed; therefore, the microscopic characteristics and mechanisms underlying the occlusions could not be determined.

The patency findings should therefore be interpreted descriptively. The absence of a between-group difference in this sample does not establish equivalence or non-inferiority.

## 4. Discussion

The principal finding of this exploratory controlled animal study was a large reduction in reconstruction time with IMAS. Mean anastomosis time decreased from 20 min 32 s with conventional interrupted microsurgical suturing to 5 min 30 s with IMAS, a difference of 15 min 02 s (95% CI, 14 min 19 s–15 min 45 s) and an approximately 73% reduction. The observed patency proportions were 95% in both groups at 1 week and 90% in both groups at 1 month; these proportions are descriptive and should not be interpreted as evidence of equivalence.

The time reduction is mechanistically plausible because the timed portion of conventional reconstruction consists of repeated transmural needle passage, placement of multiple interrupted sutures, and knot tying around the vessel circumference. In contrast, the IMAS procedure substitutes intraluminal positioning of the two vessel ends followed by two external circumferential ligatures.

The study design also helps clarify what contributed to the timing difference. Anesthesia, laparotomy, vessel exposure, heparinized irrigation, clamp placement, and other common preparatory steps occurred before complete transection and were therefore outside the timed interval. Likewise, wound closure occurred after reperfusion. The measured interval was restricted to reconstruction between transection and restored flow, using the same endpoints in both groups.

The circumferential ridge is the principal retention element of IMAS. A smooth intraluminal tube can maintain alignment yet still permit longitudinal slippage of a vessel end. In IMAS, the vessel is advanced over the ridge and then compressed externally by the ligature. The intended mechanical effect is therefore resistance to withdrawal rather than depth control alone.

This ridge-and-ligature concept distinguishes IMAS from a simple luminal splint. It also avoids the repeated wall penetration of conventional interrupted suturing, although it introduces a permanent intraluminal foreign body and direct ridge–intima contact, both of which create separate biological and hemodynamic concerns.

The IMAS mechanism is also distinct from classical cuff techniques used in experimental transplantation and vascular graft models [5,6]. Cuff techniques can substantially reduce reconstruction time but generally require the vessel to be placed or everted over a cuff and secured around it. In the present IMAS procedure, the vessel ends were advanced directly over an intraluminal microtube without eversion and were retained by the internal ridge plus external ligature.

Continuous suturing is another time-saving strategy, but it remains fundamentally needle- and tension-dependent [2,3,4]. It reduces the number of knots relative to interrupted suturing but does not eliminate repeated transmural needle passage. IMAS instead changes the fixation method itself from circumferentially distributed transmural sutures to two ridge-supported circumferential ligatures.

The previously cited stent-assisted literature also provides important context [7,8,9,10]. Senthil-Kumar et al. used a hollow intraluminal stent primarily as luminal support for photochemical tissue bonding and reported loss of patency in several stent-containing repairs at 1 week, emphasizing the thrombogenic implications of a retained intraluminal device [7]. Saegusa et al. used an expandable NiTi microstent combined with adhesive fixation and incorporated thrombus, histological, and vessel-strength assessments [8]. Aizawa et al. used an intravascular stent to support the lumen during cyanoacrylate bonding rather than as the definitive ridge-based retention mechanism [10], while modified microstent approaches have also been explored in vivo [9]. These systems differ from IMAS in their principal fixation method: the cited approaches rely on luminal support, expansion, or adhesive bonding, whereas IMAS relies on bilateral circumferential ridges mechanically captured by external ligatures. The present study did not directly compare these alternative systems and does not establish superiority over them.

Because all operations in both groups were performed by the same surgeon, who was already proficient in conventional microvascular suturing, the time difference cannot be attributed to different surgeons assigned to the two techniques. In addition, 10 pilot IMAS procedures were completed before the formal experiment to reduce the influence of initial device-handling and technique familiarization. This single-operator design reduced interoperator variability and made the comparison internally consistent within this laboratory setting.

However, several potential sources of bias remain. Permuted block randomization with a fixed block size of two maintained balanced group allocation, but it also introduced some predictability. The first assignment within each block was concealed until immediately before the procedure; however, once that assignment had been revealed, the second assignment in the block was fully predictable. Thus, allocation concealment effectively applied only to the first animal in each block. In addition, although treatment order was randomized within blocks, the sequence did not guarantee strict alternation across the entire experiment, and identical procedures could occur consecutively at block boundaries. The operating surgeon necessarily knew which technique was being performed, and the stopwatch observer was also not blinded because the reconstruction technique was directly visible. Video-based independent timing was not used. These limitations are particularly relevant because anastomosis time was the primary outcome.

The patency findings require a different interpretation from the procedural-time endpoint. The observed proportions were identical in the two groups at both scheduled follow-up points, but a non-significant Fisher exact test in 20 animals per group does not demonstrate equivalence. With only one or two occlusions per group, the estimates are imprecise for detecting modest differences in failure risk.

No prospective sample-size calculation was performed, and no non-inferiority margin was specified. The study therefore had a clear signal for the large procedural-time difference but was not designed to establish similarity in patency. The appropriate conclusion is that IMAS shortened anastomosis time while producing observed 1-week and 1-month patency proportions of 95% and 90%, respectively, in this sample.

A permanent intraluminal implant raises safety questions that binary patency alone cannot resolve. Contact between the ridge and intima may cause endothelial injury; the resin surface may influence platelet adhesion or thrombogenicity; and altered local geometry may affect shear conditions and flow separation.

The prior stent literature reinforces the importance of these endpoints. Studies of alternative intraluminal systems have incorporated histological assessment, thrombus evaluation, vessel strength, or short-term tissue response [7,8,10]. In the present study, these complementary measurements were not performed, limiting the ability to distinguish a mechanically patent vessel from one with subclinical wall injury, thrombus, or progressive neointimal change.

Accordingly, Doppler-detectable flow at 1 month should not be interpreted as evidence of normal endothelial structure or long-term vascular safety. Histology is needed to evaluate endothelial continuity, inflammatory reaction, thrombus organization, fibrosis, and neointimal thickness at the ridge and tube interfaces.

Device geometry also requires further optimization. The verified dimensions used in the formal study were 1.6 mm inner diameter, 1.8 mm outer diameter, and 8.5 mm overall length. This size was selected after preoperative ultrasonographic screening of the abdominal aorta in 250–300 g male Sprague Dawley rats and pilot testing of three adjacent device sizes. During the 10 pilot IMAS procedures, devices with inner/outer diameters of 1.4/1.6 mm, 1.6/1.8 mm, and 1.8/2.0 mm were evaluated; the 1.6/1.8 mm device provided the most suitable practical fit and was used in the formal experiment. Because the ultrasonographic screening was performed for device sizing rather than as a prespecified quantitative endpoint, cohort-level vessel-diameter data were not analyzed. Future studies should prospectively quantify vessel caliber and the device-to-vessel diameter relationship and determine whether relative oversizing or undersizing influences intimal injury, stenosis, thrombosis, or technical feasibility.

Mechanical security was assessed only indirectly by gross retention of the device and the absence of obvious leakage or aneurysmal dilatation in patent vessels. The study did not measure pullout force, tensile resistance, or burst pressure. These tests are necessary to quantify the contribution of the anchoring ridges and to compare mechanical strength directly with sutured anastomoses.

Quantitative hemodynamic assessment is likewise needed. Color Doppler ultrasonography was used to identify flow and to compare proximal and distal aortic luminal caliber qualitatively, and terminal longitudinal opening of the aorta allowed gross inspection for intraluminal narrowing. However, peak velocity, flow volume, turbulence, calibrated luminal diameter, and percentage stenosis were not quantified. Such data would be particularly important because the 1.6 mm internal lumen and the ridge–vessel interface may alter local flow even when gross patency is preserved.

Material characterization remains an important limitation. The implanted material can now be identified as Anycubic Tough Ultra Resin (Anycubic, China), and the manufacturer-provided technical specification reports basic photopolymer and mechanical properties, including 365–405 nm curing compatibility, density 1.05–1.25 g/cm3, Shore hardness 71–75 D, tensile strength 20–28 MPa, elongation at break 70–80%, flexural strength 15–25 MPa, and flexural modulus 350–800 MPa. These technical properties, however, do not constitute evidence of suitability for permanent blood-contacting implantation. The retained study record does not document the lot number, detailed chemical composition, formal medical-device biocompatibility classification, study-specific post-curing parameters, sterilization method, surface finishing, manufacturing tolerances, or dedicated hemocompatibility testing. These missing data limit reproducibility and prevent a definitive assessment of material-related vascular safety.

For these reasons, the present results should be viewed as a proof-of-feasibility evaluation of the fixation concept rather than a definitive safety study of a permanent vascular implant. Before translational use, the material and manufacturing process would require formal characterization and appropriate biocompatibility and hemocompatibility testing.

The study has several additional limitations. First, follow-up was limited to 1 month, which cannot establish long-term durability, late stenosis, chronic inflammation, or progressive neointimal hyperplasia.

Second, histological analysis, quantitative stenosis measurement, quantitative flow assessment, and mechanical strength testing were not performed. The underlying causes and microscopic mechanisms of the non-patent cases therefore remain undetermined.

Third, the experiment used only male SPF Sprague Dawley rats. Although selection of male animals reduced procedural complexity and anatomical variability and facilitated standardized microsurgical exposure in this feasibility model, the findings may not generalize to female animals or other strains. In addition, a detailed prespecified definition of humane endpoints was not available for retrospective reporting.

Fourth, the single-surgeon design reduces between-operator confounding but limits generalizability to other surgeons and does not establish whether IMAS shortens the learning curve across operators. Although the surgeon completed 10 pilot IMAS procedures before the formal experiment, the learning curve beyond this initial familiarization was not formally quantified. In addition, non-blinded stopwatch measurement may have introduced measurement bias.

Fifth, the sample size was exploratory and not based on a prospective effect-size calculation. The observed anastomosis-time difference was large and was estimated with a relatively narrow confidence interval in this dataset; however, the sample was not designed to detect clinically or biologically meaningful differences in patency or to estimate uncommon adverse events with precision.

Despite these limitations, the experiment demonstrates that bilateral ridge-based intraluminal retention combined with two external ligatures can be used to reconstruct a completely transected rat abdominal aorta and can markedly reduce reconstruction time under the conditions tested.

The short-term patency observations support continued investigation of the concept but should not be interpreted as proof of equivalence, long-term safety, or readiness for clinical application.

Future studies should consider randomization schemes using variable block sizes to reduce allocation predictability and should use blinded or independently adjudicated assessment of procedural time where feasible. Video recording of procedures would allow objective verification of the primary timing endpoint and formal analysis of any residual learning-curve effect.

Histological evaluation should assess endothelial continuity, inflammatory cell infiltration, thrombus formation and organization, fibrosis, and neointimal thickness, with particular attention to the vessel wall overlying the anchoring ridges.

Mechanical testing should include burst pressure and axial pullout or tensile resistance to quantify anastomotic security and define the contribution of ridge geometry and ligature tension.

Quantitative vascular imaging should include vessel diameter before implantation and serial measurements of effective lumen, percentage stenosis, peak or mean flow velocity, and, where possible, flow disturbance across the reconstructed segment.

Future material studies should report the resin lot number, detailed composition or relevant safety documentation, study-specific post-curing and sterilization processes, surface finish, dimensional tolerances, and appropriate blood-contacting biocompatibility and hemocompatibility data. Alternative materials or surface treatments may be required if thrombogenicity or chronic wall reactions are identified.

Larger cohorts and longer follow-up are necessary to estimate patency and complication rates with greater precision. Future designs should be powered separately for the procedural-time endpoint and for any patency non-inferiority or equivalence hypothesis, with a clinically justified margin defined in advance.

Studies involving multiple surgeons with different levels of microsurgical experience would determine whether the observed procedural simplification is reproducible across operators and whether it alters learning curves, technical-failure rates, or interoperator variability.

Finally, evaluation in larger animal models and vessels of different calibers will be necessary before clinical translation can be considered. At present, IMAS should be regarded as an experimental fixation concept with a strong procedural-time signal but incompletely characterized biological, hemodynamic, mechanical, and material safety.

## 5. Conclusions

In this rat infrarenal abdominal aortic model, IMAS reduced mean anastomosis time from 20 min 32 s to 5 min 30 s, an approximately 73% reduction. The estimated mean difference was 15 min 02 s (95% CI, 14 min 19 s–15 min 45 s) under a standardized single-surgeon protocol with identical timing endpoints.

Observed patency was 95% in both groups at 1 week and 90% in both groups at 1 month. These descriptive findings do not establish equivalence or non-inferiority. Because the device remains permanently intraluminal and the present study did not evaluate histological response, quantitative stenosis or flow, mechanical strength, detailed material biocompatibility, or long-term outcomes, further controlled studies are required before the safety or broader applicability of IMAS can be determined.

## Figures and Tables

**Table 1:** Operative and vascular outcomes. SD, standard deviation; IMAS, intraluminal microtube anastomosis system. The mean difference in anastomosis time (conventional minus IMAS) was 902 s (95% CI, 859–945 s). Patency *p* values were calculated using two-sided Fisher’s exact tests because the 2 × 2 contingency tables were sparse, with only one or two non-patent vessels per group at the scheduled follow-up points.

Outcome	IMAS (*n* = 20)	Conventional (*n* = 20)	*p*-Value
Anastomosis time, mean ± SD	5 min 30 s ± 60 s	20 min 32 s ± 75 s	<0.001
Patency at 1 week	19/20 (95%)	19/20 (95%)	1.000
Patency at 1 month	18/20 (90%)	18/20 (90%)	1.000

## Data Availability

The detailed research data supporting the findings of this study are not publicly available due to intellectual property considerations related to the potential patenting and commercialization of the IMAS device.
